# Differences in Virulence among PVY Isolates of Different Geographical Origins When Infecting an Experimental Host under Two Growing Environments Are Not Determined by HCPro

**DOI:** 10.3390/plants10061086

**Published:** 2021-05-28

**Authors:** Mongia Makki, Francisco Javier del Toro, Khouloud Necira, Francisco Tenllado, Fattouma Djilani-Khouadja, Tomás Canto

**Affiliations:** 1 Laboratory of Molecular Genetics, Immunology and Biotechnology, Faculty of Sciences of Tunis, University of Tunis El Manar, Manar II, Tunis 2092, Tunisia; mongiamaki@gmail.com (M.M.); necirakhouloud@gmail.com (K.N.); fattouma.djilani@yahoo.fr (F.D.-K.); 2 Department of Microbial and Plant Biotechnology, Margarita Salas Center for Biological Research, Spanish Council for Scientific Research (CIB Margarita Salas-CSIC), Ramiro de Maeztu 9, 28040 Madrid, Spain; fjdts@cib.csic.es (F.J.d.T.); tenllado@cib.csic.es (F.T.)

**Keywords:** HCPro and virus adaptation, *Potyvirus* virulence and HCPro, HCPro suppression of silencing, *Potyvirus* evolution

## Abstract

The contribution of the HCPro factors expressed by several PVY isolates of different geographical origins (one from Scotland, one from Spain, and several from Tunisia) to differences in their virulence in *Nicotiana benthamiana* plants was investigated under two growing conditions: standard (st; 26 °C and current ambient levels of CO_2_), and climate change-associated (cc; 31 °C and elevated levels of CO_2_). In all cases, relative infection symptoms and viral titers were determined. The viral *HCPro* cistrons were also sequenced and amino-acid features of the encoded proteins were established, as well as phylogenetic distances. Additionally, the abilities of the HCPros of several isolates to suppress silencing were assessed under either growing condition. Overall, viral titers and infection symptoms decreased under cc vs. st conditions. However, within each growing condition, relative titers and symptoms were found to be isolate-specific, with titers and symptom severities not always correlating. Crucially, isolates expressing identical HCPros displayed different symptoms. In addition, all HCPro variants tested displayed comparable silencing suppression strengths. Therefore, HCPro alone could not be the main determinant of the relative differences in pathogenicity observed among the PVY isolates tested in this host, under the environments considered.

## 1. Introduction

In compatible infections, viruses overcome antiviral defenses to spread systemically from the point of infection. To remain in an environment, viruses also need to further disperse to other plants. Horizontal dispersal of plant viruses can follow different strategies, but the vast majority of viruses have co-evolved mechanisms of transmission with specific insects as vectors [1,2,3].

In the case of viruses of the genus *Potyvirus*, the multifunctional, non-structural protein helper component protease (HCPro) [4] has been known, for a long time, to be a pathogenicity determinant: mutations in this protein had the ability to reduce viral titers and attenuate symptoms, and even to prevent viruses from infecting plants systemically [5,6,7]. These observations on HCPro as a determinant of pathogenicity were understood better when they were associated with the silencing-suppression function of this protein [8]. Thus, in a collection of lab-generated Tobacco etch virus HCPro mutant variants, their different relative silencing suppression strengths affected virulence in *Nicotiana benthamiana* plants: decreased silencing suppressor activities correlated with reduced symptoms and titers, but increased silencing suppressor activities did not lead to neither increased symptoms nor titers, relative to non-mutant viruses [9].

In addition to determining pathogenicity and suppressing silencing, HCPro has also been shown to intervene, along with virions, in the non-persistent transmission of infection by aphid vectors [10,11,12,13]. Interestingly, the non-persistent transmission of the potyvirus *Potato virus Y* (PVY) by aphids strongly correlates with viral titers in the leaves of the infected donor plant [14,15]. HCPro intervenes thus in two ways in the horizontal dispersal of potyviral infections in nature: on the one hand, directly, by participating mechanistically in the transmission process; on the other hand, indirectly, by helping virions and HCPro reach the titers in infected aerial mesophyll and epidermal cells that are required for an efficient transmission of infection by the insect vectors that probe or feed on them. 

HCPro therefore helps potyviruses accumulate and spread systemically in their compatible hosts, and also between hosts. It could be hypothesized that in a given compatible host plant and growing environment, HCPro might be a major player at regulating viral titers in the infected cells of systemic leaf tissues, to secure efficient transmission by vectors, without causing such damage to host populations through their virulence as to jeopardize the permanence of the virus in that environment [16,17]. In this regard, computational analysis of nucleotide variations among potyvirus genomes has shown the presence of hyper-variable regions in the cistrons of several virus-encoded factors, including *HCPro*, where non-synonymous substitutions could take place under positive selection, allowing room for the occurrence of genetic adaptation of viral isolates to specific hosts, as well as for the modulation of their virulence [18], but at the expense of their genetic robustness to alterations to such environments [19].

We have assessed whether HCPro encoded by potyvirus isolates originating from different climates could possess differential adaptations to facilitate efficient systemic infection and dispersal in those environments. Here, we have compared infections by PVY isolates of different geographical origins and climates of a common compatible host (*N. benthamiana*), under two experimental growing conditions. We have characterized the viral titers and symptoms induced by each isolate in this host under both growing conditions, as well as molecular features of their expressed HCPro proteins, including relative silencing suppression strengths. With these data, we have assessed how determinant HCPro is to differences in infection outcomes observed in this host, under the environments considered. 

## 2. Materials and Methods

### 2.1. Experimental Plants and Their Growing Conditions

*N. benthamiana* plants were used as a common compatible host of the virus isolates used in this study. Plants were kept in environment-controlled growth chambers with a 16/8-h day/night photoperiod and ~2500 lux of daylight intensity. Two 24-h round-the-clock environment conditions of temperature and CO_2_ levels were used: standard conditions (st) of 26 °C and current atmospheric CO_2_ levels (of around 405 parts per million, ppm); conditions of simultaneously elevated temperature and CO_2_ levels of 31 °C and CO_2_ 970 ppm (hereafter, climate change-associated conditions, cc). 

### 2.2. Virus Isolates

A collection of PVY isolates from different geographical origins were used in this work: an aphid-transmissible Scottish isolate from potato of the PVY^O^ common strain group (PVY^O^ SCRI-O) described by Barker et al. [20]; a non-aphid-transmissible isolate, also of the PVY^O^-strain group, obtained from pepper plants in Southern Spain (PVY 1-2, isolate P-22-88) [21,22]; and several isolates that were sampled from field-grown crops in the month of August, at the start of the main potato growing season, in two separate locations in Tunisia: one site by the coast (Monastir, Lat. 35.7345/Long. 10.8126; isolates 28, 41, 43, 53, 59A, M2, M5D, SP, X1, all of them in the PVY^NTN^ group; plus isolate MIE, of PVY^NW^-type), and another site in the interior of the country (El Kef, Lat. 36.0357/Long. 8.7249; isolates Kef K2, Kef M, Kef Pep2, Kef 7, Kef 11, all of them in the PVY^NTYN^ group). All of the Tunisian isolates were sampled from potatoes (Spunta variety), with the exception of isolate Kef Pep2 that was sampled from pepper, and were group-characterized according to Chik Ali et al. [23] (data not shown). The isolates used in this work originated from four sites in three countries with distinct climates (Figure S1). 

The Scottish and Spanish PVY isolates had previously been maintained in *Nicotiana* plants in the lab. In the case of the Tunisian isolates, a *N. benthamiana* plant was mechanically inoculated in the lab with extracts from symptomatic leaves sampled in field trips from potato and pepper plants, made in ice-cold 0.1 M Na-phosphate buffer, pH 8 (1/1, *w*/*v*). This single-passage, infected *N. benthamiana* plant became the common source for any further experimentation. To this purpose, symptomatic systemic *N. benthamiana* leaves from this source plant were ground in phosphate buffer as described above, and the resulting extract was aliquoted and kept frozen at −80 °C until further use as source of inoculum. Extracts from the source plants were confirmed for the presence of PVY and for the absence of PVX or CMV by Western blot, before further experimentation.

### 2.3. Characterization of the HCPro Cistrons of the Viral Isolates, and Their Cloning 

To amplify and clone the *HCPro* cistrons of the PVY isolates used in this study, we first generated cDNAs from total RNAs extracted from the corresponding infected *N. benthamiana* source plants, using a reverse oligo complementary to a region of the viral RNA downstream the *HCPro* cistron that is extremely conserved among the available PVY sequences (oligo P3′RT; Table 1) and SuperscriptIII DNA polymerase, following the manufacturer’s instructions (Invitrogen, Carlsbad, CA, USA). 

Amplification by PCR of the *HCPro* cistrons from cDNAs was performed using high-fidelity Phusion^Tm^ DNA polymerase (Invitrogen). Oligos specific for the Scottish and Spanish accessions were designed (Table 1) using their already known nucleotide HCPro sequences [20], GenBank accession number AJ585196.1 of the whole viral sequence and [21], GenBank accession number AF166115, respectively. For the Tunisian isolates, degenerate oligos were used, which once the fragments had been cloned, turned out to have the sequences shown in Table 1. To allow the cloning into plasmids of the amplified PCR fragments, the forward oligos had an added *BamH*I site at their 5′-end. Because the *HCPro* cistron is translated as part of a polyprotein that undergoes post-translational cleavage, downstream of the *BamH*I restriction site, two triplets—ATG GCA (Met, Ala)—were added upstream of and in frame with the first HCPro Ser-encoding triplet, in order to facilitate transient protein expression in plants from binary constructs through improved Kozak translation consensus, as described by Tena et al. [24]. The reverse oligos had an added stop codon downstream of the HCPro ORF, followed by a *Sac*I site in the case of the Spanish and Scottish isolates, and by a *Kpn*I site in the case of the Tunisian isolates, as it turned out that *Sac*I was already present inside the Tunisian HCPro sequences and this restriction site had to be replaced by that of *Kpn*I. 

**Figure 1:**
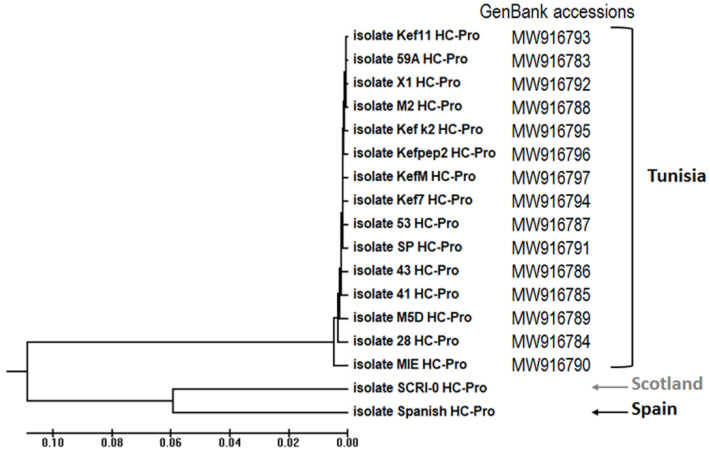
Phylogenetic analysis of the 1368 nucleotide-long sequence of the *HCPro* cistrons encoded by the 17 isolates of potato virus Y (PVY) of different geographical origins (Scotland, Spain and two separate sampling sites in Tunisia: Monastir and El Kef) used in this work. The GenBank accessions of the Tunisian isolates are indicated. The phylogenetic UPGMA tree was generated using the Mega6 program after alignment of sequences of HCPRO with ClustalW software. The analyzed isolates are grouped into three phylogenetic branches, whFich followed their countries of origin.

PCR fragments were cloned into the high-copy intermediate plasmid pBluescript SK^2+^ and from there, some were transferred into low-copy pROK2 binary vectors, in both cases, after digestion with either BamHI/SacI or *BamH*I/*Kpn*I. Alternatively, some were cloned directly into the binary vector in the same way. Sequencing was performed by Secugen SA (Madrid, Spain) using sequencing oligos from the plasmids that flanked the cloned inserts. Contigs of the sequences obtained were assembled that covered the whole cloned *HCPro* cistrons, and their encoded amino acid sequences were also deduced, using Vector NTI^®^ software (Invitrogen). The HCPro sequences of the Tunisian isolates were deposited in GenBank (accession numbers are shown in Figure 1). Phylogenetic analysis of the HCPro sequences was performed using ClustalW software (http://www.clustal.org/ (accessed on 15 April 2021), Dublin, Ireland).

### 2.4. Relative Quantitation of Proteins Expressed Transiently by Agroinfiltration, and of Systemic Viral Titers in Infected Plants

Relative viral titer quantitation was performed using four plants per virus isolate in each experiment. Quantitation was doubly performed: by RT-qPCR, measuring genomic RNA levels, and by Western blot, measuring viral coat protein (CP) levels. 

Genomic RNA levels were determined by one-step reverse transcription plus real time quantitative polymerase chain reactions (RT-qPCR). Briefly, total RNA was extracted from three pooled discs of 1 cm diameter each of systemic leaf tissue for each tested plant using TRIzol reagent (Invitrogen) following the manufacturer’s instructions, and contaminant DNA was removed by treatment with a TURBO DNA-free kit (Ambion, Austin, TX, USA) as described [25,26]. RT-qPCR was performed using a final reaction volume of 15 uL that contained 7.5 μL of Brilliant III Ultra-Fast RT-qPCR Master Mix (Agilent, Santa Clara, CA, USA), 1.8 μL of RNase-free water, 0.75 μL of reverse transcriptase (Agilent), 0.15 μL of 100 mM dithiothreitol (Agilent), 0.3 μM each primer, and 3 μL of total RNA extract (approximately 15 ng RNA/μL). Relative quantifications were calculated by the ΔΔCt method. 

Three pairs of oligos were specifically designed and tested for the qPCR amplification of all the PVY isolates. However, in trial tests, none of the three pairs could successfully amplify all of the isolates from the three countries used in this work. Therefore, for the amplification of the Scottish and Spanish isolates, the following pair was used: qPCR-Scot-Spain Fw and qPCR-Scot-Spain Rv (Table 1). For the amplification of the Tunisian isolates, the following pair was used: qPCR-Tunis Fw and qPCR-Tunis Rv (Table 1). In addition, oligos 18SrRNA-Fw and 18SrRNA-Rv for 18S rRNA amplification were used for normalization in all cases (Table 1). RT-qPCRs were performed in a Rotor-Gene Q thermal cycler (Qiagen, Venlo, The Netherlands) using the following thermal protocol: 50 °C for 10 min; 95 °C for 3 min; 40 cycles of 95 °C for 10 s and 60 °C for 20 s; and a final ramp for melting analysis from 60 °C to 95 °C, rising 1 °C every 5 s. 

Relative systemic viral titers were also determined by measuring CP levels by Western blot. Total proteins were extracted from three pooled discs of 1 cm diameter of systemic leaf discs/plant as described [26]: briefly, discs were mechanically ground in ice-cold extraction buffer (0.1 M Tris-HCl PH 8, 10 mM EDTA, 0.1 M LiCl, 1% β-mercaptoethanol and 1% SDS), boiled for 5 min, clarified by bench centrifugation and fractionated in 15% SDS-PAGE gels, and transferred by Western blot to PVDF membranes. To detect and quantify the viral CP, a rabbit polyclonal antiserum to the PVY CP was used [21]. A commercial alkaline phosphatase-labeled goat anti-rabbit secondary antibody (Sigma-Aldrich, San Luis, MO, USA) and BCIP/NBT substrate solutions (Duchefa) were also used. Western blot membranes were scanned, and relative band densities were quantified using Image J software (v. 1.48; www.imagej.nih.gov (accessed on 15 April 2021), National Institute of Mental Health, Bethesda, MD, USA).

### 2.5. Assessment of Infection Symptoms in Plants Grown under st or cc Conditions

*N. benthamiana* plants of the same growing batch were mechanically inoculated with extracts from plants infected with each of the PVY isolates studied, using the frozen aliquots obtained from the corresponding *N. benthamiana* source plants. In each experiment and for each isolate, four same-age, ten-day-old plants were rub-inoculated onto three consecutive carborundum-dusted leaves (25 µL of extract/leaf). Inoculated leaves were rinsed with distilled water immediately afterwards, and plants were transferred to either st or cc growing conditions. Ten days after inoculation, plant symptoms were visually assessed for symptoms and classified into four ranges of pathogenicity (i.e., in their ability to elicit symptoms in this experimental host): mild (no leaf curling, no plant stunting, some mosaic); intermediate (minor leaf curling and stunting; mosaic); strong (severe leaf curling and stunting); very strong (severe leaf curling and stunting, presence of necrosis). Samples from non-necrotized systemic leaf tissue were taken for the measure of viral titers, as is described further below. 

### 2.6. Silencing Suppression Agropatch Assays

HCPro variants cloned into binary vectors were transiently expressed in mesophyll and epidermal cells of fully expanded leaves of *N. benthamiana* plants using the agroinfiltration technique, to test for their relative abilities to suppress the silencing of a transiently co-expressed GFP reporter (the HCPros of the Scottish and Spanish isolates, and of Tunisian isolates 41, 59A, MIE and Kef Pep2 were tested in this way). Briefly, agrobacterium cell cultures carrying binary constructs were grown exponentially at 28 °C in liquid LB media under shaking, with the appropriate antibiotic selection (kanamycin resistance provided by the pROK2-based vectors, and rifampicin), pelleted, and re-suspended to an optical density at 600 nm of 0.3 in 2-N-morpholino ethanesulfonic acid (MES)-based infiltration buffer containing acetosyringone 0.2 mM to activate T-DNA transfer. After incubation at room temperature for 2 h, bacterial solutions were co-infiltrated together (1/1 *v*/*v* mixtures, each at OD 600 of 0.3) with bacterial solutions containing either empty pROK2 vector, or a vector that expressed the reporter GFP, as described [27]. 

In agropatch assays, transient expression in plant cells from T-DNAs of binary vectors delivered by agroinfiltration was carried out under either st or under cc conditions as described [25]. In the former case, agroinfiltrations were performed under st conditions, and plants were kept under those conditions for a further four days. In the latter case, agroinfiltrations were also performed under st conditions, and plants were kept in those conditions for the first 24 h, to allow for the effective transfer of T-DNA from bacteria to plant cells, followed by a further 3 days at cc conditions. In both cases, steady-state levels of GFP were then assessed under a UV lamp (Black Ray long wave UV lamp; UVP) and photographed exactly under the same illumination and camera set conditions, to allow direct comparisons between leaves, and leaf discs from the infiltrated patches were collected for the relative quantification of the steady-state levels of the GFP reporter by Western blot, as described already. 

Total proteins were extracted from agroinfiltrated leaf discs and fractionated in SDS-PAGE gels (15% polyacrylamide gels). Resolved proteins in gels were then wet-transferred (Western blot) to PVDF membranes (Amersham-GE Healthcare, Chalfont, UK) as described [28]. To detect the GFP band, a polyclonal rabbit antiserum donated by G. Cowan (The James Hutton Institute, Dundee, UK) was used. A commercial alkaline phosphatase-labeled goat anti-rabbit secondary antibody (Sigma-Aldrich) and BCIP/NBT substrate solutions (Duchefa, Haarlem, NL) were also used.

## 3. Results

### 3.1. Characterization of the HCPro Sequences Encoded by the PVY Isolates Used in this Work

The nucleotide sequences of the *HCPro* cistrons of all 17 PVY isolates were determined and compared. Those from the Scottish and Spanish isolates resulted in five and two amino acid substitutions in the encoded protein, relative to those expected from the published sequences AF166115 and AJ585196.1, respectively: Scottish HCPro had amino acid substitutions at positions 109 (TCG, Ser → T**T**G, Leu), 184 (CCT, Pro → **T**CT, Ser), 240 (CTA, Leu → C**C**A, Pro), 290 (CGT, Arg → **T**GT, Cys), 349 (ACA, Thr → **G**CA, Ala); Spanish HCPro had amino acid substitutions at positions 90 (CTC, Leu → **T**TC, Phe), and 407 (GTT, Val → **A**TT, Ile). No additional silent substitutions were found in either sequence. The observed differences could derive from errors in the initially published sequences derived from the use of Taq polymerases of low fidelity in the RT-PCR amplification and/or from manual sequencing, or alternatively, from substitutions arising in these isolates after the time of their initial sequencing and publication, as they may have become more adapted to *Nicotiana* plants through passages under standard laboratory conditions, since they were initially sampled.

The complete *HCPro* cistrons of the fifteen Tunisian isolates were sequenced de novo after first mechanical passage into *N. benthamiana* source plants (se Methods Section) and their corresponding GenBank accession numbers are shown in Figure 1. None of the sequences were identical among each other at the nucleotide level, although they, nevertheless, showed high identity percentages. When the seventeen sequences were compared, all the Tunisian isolates clustered phylogenetically into one single group, independently of the geographic sites in the country from which they originated, separated from the Spanish and Scottish isolates, which branched separately (Figure 1). 

**Figure 2:**
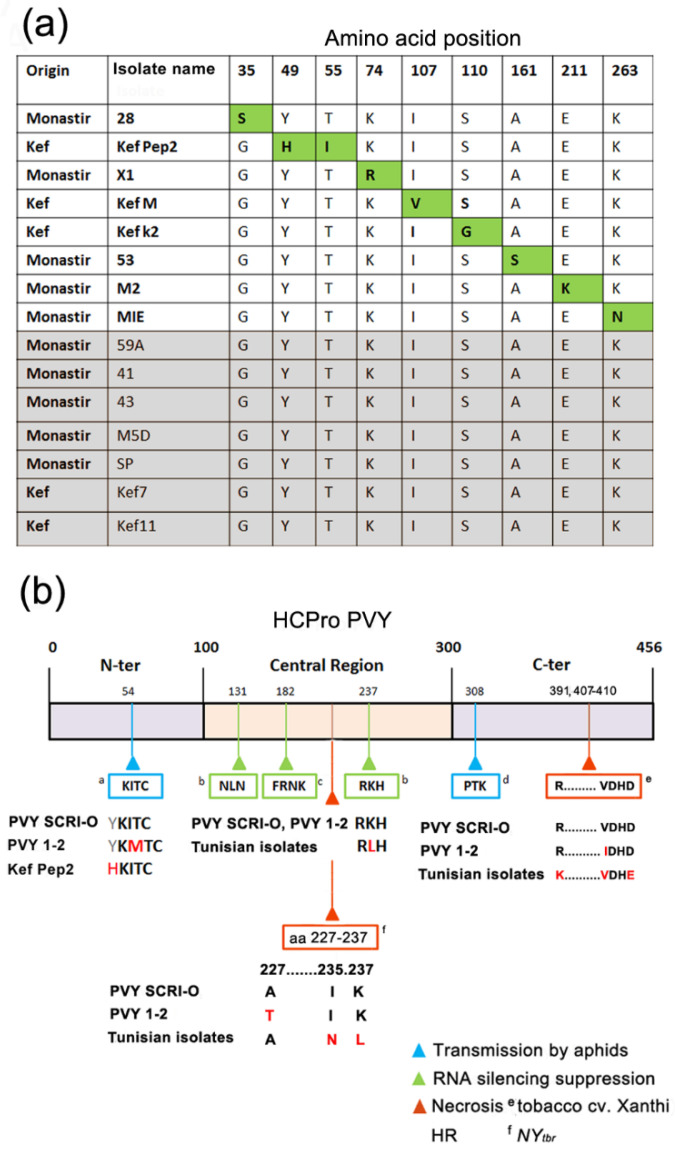
(**a**) Analysis of differences in the amino acid sequences encoded by the *HCPro* cistrons of the fifteen isolates of potato virus Y (PVY) from Tunisia. Their geographical origin (Monastir or El Kef) is indicated in first column. In the case of eight of the isolates, differences with the other isolates were limited to only one amino acid (isolates 28, X1, Kef M, Kef K2, 53, M2, MIE) or in one case, to two amino acids (isolate Kef Pep2), which appear highlighted in green. The HCPro amino acid sequences of seven of the isolates were identical to each other (highlighted in grey; isolates 59A, 41, 43, M5D, SP, Kef 7, Kef 11). (**b**) Schematic representation of the 456 amino acids of the HCPro protein, and of some published domains of relevance to its function in the aphid-mediated transmission of infection and in the suppression of antiviral silencing and how they relate to the sequences of the isolates used in this work: ^a^ [5]; ^b^ [28]; ^c^ [7,29]; ^d^ [30]; ^e^ [31]; ^f^ [32].

At the amino-acid level, differences among the Tunisian isolates were even smaller. In fact, the HCPro of seven of the isolates was identical, even though some of them were sampled in different geographical regions. For example, isolates 41 and 59A expressed the same protein, although their *HCPro* cistrons had five silent nucleotide differences (Figure 2a). The eight remaining HCPro proteins all differed from one another, but differences were limited to only one, and in one case to two, amino acids (Figure 2a), out of the 456 of the protein. On the other hand, amino acid differences between the Tunisian HCPros and the Scottish or Spanish HCPros were numerous (amino acid identities with their two European counterparts ranging from 89 to 91%; data not shown).

### 3.2. Systemic Viral Titers of the PVY Isolates in Infected Plants Kept under st or cc Conditions

**Figure 3:**
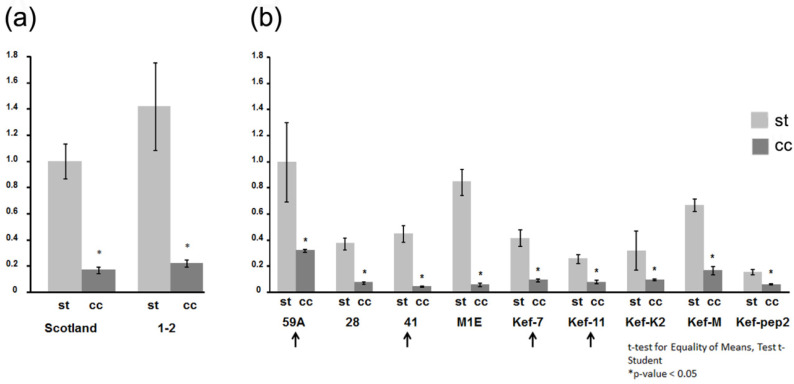
Determination of viral titers by RT-qPCR of the genomic RNA levels of the potato virus Y (PVY) isolates used in this work in systemic infected leaf tissue. Infected plants had been kept either under standard (st) growing conditions of 26 °C and current atmospheric levels of CO_2_ of ~405 parts-per-million (ppm), or under conditions of elevated temperature and CO_2_ levels (climate change-associated conditions (cc)) of 31 °C and 970 ppm of CO_2_. Relative genomic RNA titers in systemic leaves were assessed in 4 plants/isolate 10 days after their inoculation. (**a**) Relative genomic RNA titers of the Scottish and Spanish isolates obtained by RT-qPCR using oligos qPCR-Scot-Spain Fw and Rv (Table 1). Genomic RNA titers of the Scottish isolate under st conditions were given the arbitrary value of 1. (**b**) Relative genomic RNA titers of nine Tunisian isolates obtained by RT-qPCR using oligos qPCR-Tunis Fw and Rv (Table 1). Titers of isolate 59A under st conditions were given the arbitrary value of 1. Genomic RNA titers among isolates within each chart can be directly compared. For all viral isolates, genomic RNA titers in plants kept under cc vs. st conditions decreased several-fold. Arrows indicate those isolates whose HCPros are identical at the amino acid level.

We assessed relative viral titers in infected plants grown under either st or cc conditions. We assessed systemic levels of viral genomic RNA by RT-qPCR in plants infected with either the Scottish or the Spanish isolates or with any nine of the Tunisian isolates (Figure 3; Table 1). As is described in the Methods section, we had to rely on different pairs of oligos for the relative quantification of the Scottish and Spanish isolates on the one hand, and of the Tunisian isolates on the other (Figure 3a,b, respectively). For all the viral isolates assayed, genomic RNA titers in plants kept under cc vs. st conditions decreased significantly, with observed decreases ranging from 3- to 10-fold, depending on the isolate. Among the Tunisian isolates, isolates 59A and MIE displayed the smallest and largest decreases, respectively (Figure 3). Therefore, in all cases, the effect of cc conditions was a several-fold reduction in viral genomic RNA levels in systemic infected leaves, relative to st conditions. 

**Figure 4:**
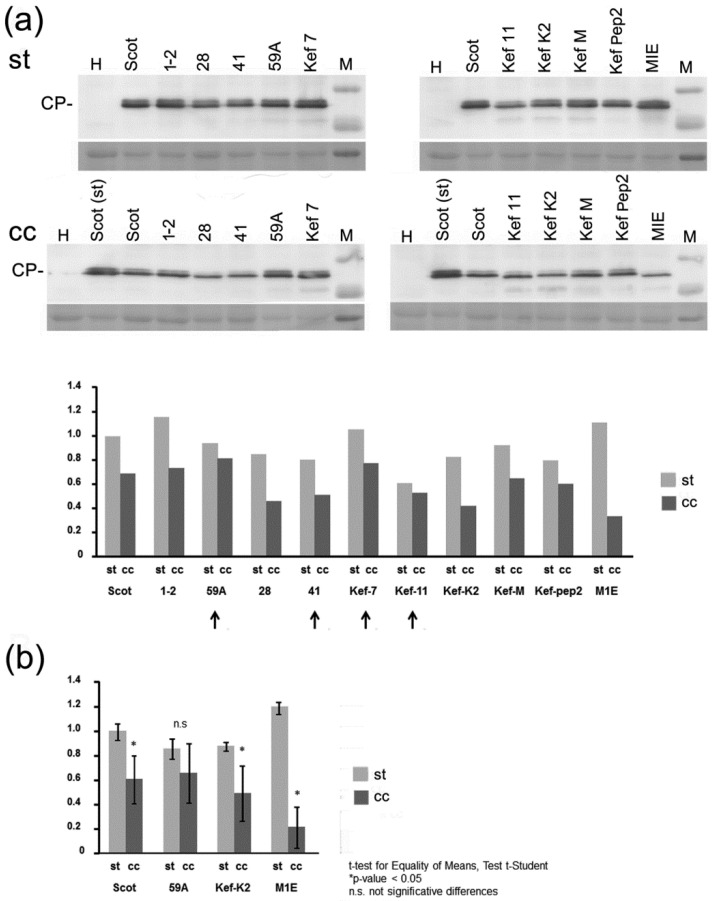
Determination by Western blot of the viral coat protein (CP) of the systemic titers of the potato virus Y (PVY) isolates used in this work. Infected plants had been kept either under standard (st) growing conditions of 26 °C and current atmospheric levels of CO_2_ of ~405 parts-per-million (ppm), or under conditions of elevated temperature and CO_2_ levels (climate change-associated conditions (cc)) of 31 °C and 970 ppm of CO_2_. (**a**) Each lane in the upper Western blot panels shows the CP levels in systemic leaves, assessed in mixtures of extracts from 4 infected plans/isolate 10 days after their inoculation. The membrane panels below the Western blot ones are the same Ponceau S-stained blotted membranes, before their incubation with antibodies, as controls of equal loading. A chart displaying the densitometry analysis of the CP bands of the Western blot is shown below. The CP titers of the Scottish isolate grown under st conditions were given the arbitrary value of 1. (**b**) Chart with the densitometry analysis of CP accumulation by Western blot in four individual plants/isolate 10 days after their inoculation, of the Scottish isolate, and of Tunisian isolates 59A, Kef Pep2, and MIE. The CP titers of the Scottish isolate grown under st conditions were given the arbitrary value of 1. CP titers in plants kept under cc vs. st conditions decreased significantly in all cases, except for isolate 59A (Student’s *t*-test, *p* < 0.05).

By Western blot, we also assessed the levels of viral CPs in the same systemic tissues in which we had measured genomic RNA levels by RT-qPCR in Figure 3. Densitometry analysis of the relative intensities of the CP bands showed that in all cases, CP levels were reduced in infected plants grown under cc vs. st conditions. However, reductions in CP titers were far less marked than those observed for the viral genomic RNAs, ranging from less than 20% (for isolate 59A) to a little over three-fold (isolate MIE) (Figure 4a). In the case of infections by isolate 59A, reductions in CP levels under cc vs. st conditions failed to be significant (Figure 4b).

### 3.3. Infection Symptoms Induced by PVY Isolates in Infected Plants Kept under st or cc Conditions

**Figure 5:**
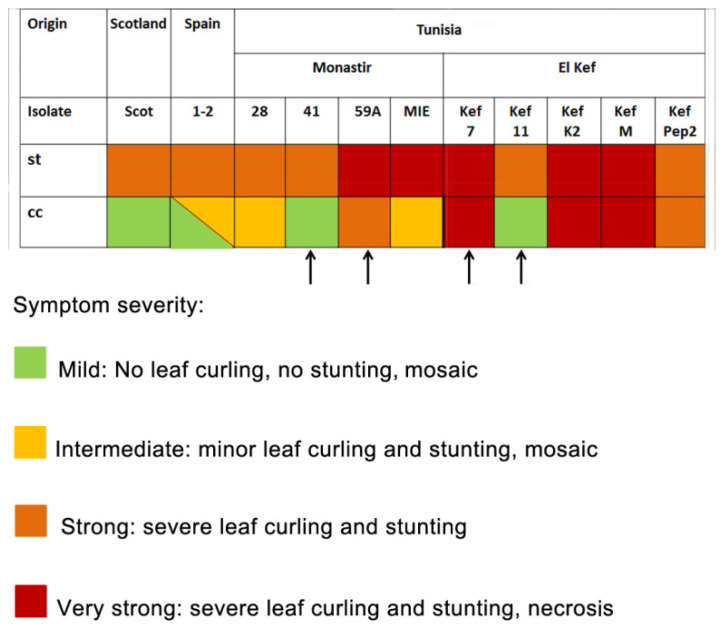
Characterization of the systemic symptoms caused by potato virus Y (PVY) isolates used in this work in the common compatible host *Nicotiana benthamiana*. Plants were kept under experimental controlled conditions of either 26 °C and current atmospheric levels of CO_2_ of ~405 ppm (standard (st) conditions), or under conditions of elevated temperature and CO_2_ levels of 31 °C and 970 ppm of CO_2_ (climate change-associated (cc) conditions). Systemic symptoms were assessed at 10 days after inoculation and could be classified into four categories: mild (no leaf curling, no plant stunting, some mosaic); intermediate (minor leaf curling and stunting; mosaic); strong (severe leaf curling and stunting); very strong (severe leaf curling and stunting, presence of necrosis). The four arrows indicate the four PVY isolates whose HCPros are identical at the amino acid level.

We compared the systemic infection symptoms induced by different PVY isolates in same-age plants of the common host *N. benthamiana*, grown under controlled st or cc conditions. We could group the symptoms caused by these infections at 10 days after inoculation into four categories, according to their severity, from milder to stronger: mild (no leaf curling, no plant stunting, mosaic); intermediate (minor leaf curling and stunting; mosaic); strong (severe leaf curling and stunting); very strong (severe leaf curling and stunting, presence of necrosis). Under st conditions symptoms for all the isolates tested were either strong or very strong (Figure 5), with severe curling and stunting as common features, in some cases also with induction of necrosis. However, under cc conditions, we observed mild, almost asymptomatic, symptoms under cc conditions for the Scottish isolate, without any leaf curling or stunting, as had been described before [26]. Mild symptoms were also recorded for the Spanish isolate, and also for some of the Tunisian isolates (41, Kef 11, which did not induce necrosis under st conditions). However, for some of the Tunisian isolates (Kef 7, K2 and M, all necrosis inducers under st conditions), symptom severity remained very strong under cc conditions (Figure 5). 

### 3.4. Relative Silencing Suppression by the HCPros Encoded by Different PVY Isolates in Agropatch Assays, under st or cc Conditions

**Figure 6:**
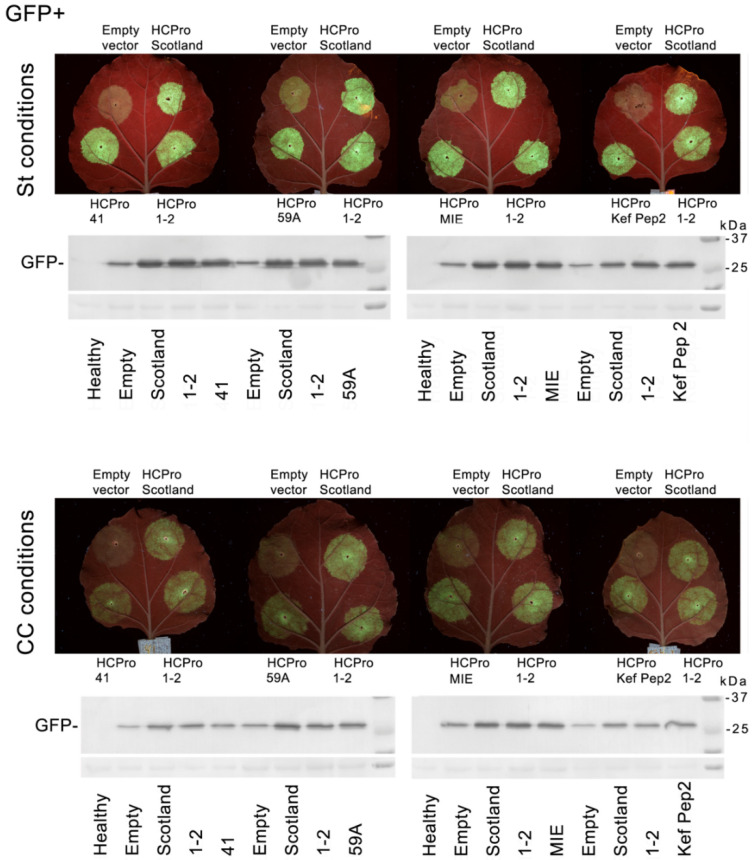
Relative silencing suppression strengths of the HCPros encoded by the potato virus Y (PVY) isolates used in this work under different growing environments. The 4-day-long agropatch assays were carried out under standard (st) growing conditions of 26 °C and current atmospheric levels of CO_2_ of ~405 ppm (upper panel of infiltrated leaves and corresponding Western blot below it), or under conditions of elevated temperature and CO_2_ levels (climate change-associated conditions (cc)) of 31 °C and 970 ppm of CO_2_ (first 24 h at st conditions, followed by 3 days at cc conditions; lower panel of agroinfiltrated leaves and corresponding Western blot below it). Bacteria harboring a binary vector expressing a GFP reporter were co-agroinfiltrated together with bacteria harboring either an empty binary vector, or binary vectors expressing Met-Ala-HCPro constructs from the PVY isolates tested in the assay.

The HCPros of six isolates (the Scottish and Spanish isolates, plus Tunisian isolates 41, 59A, Kef Pep2 and MIE) were comparatively tested in their abilities to suppress silencing in agropatch assays, under st and cc conditions. Binary constructs expressing the corresponding Met-Ala-HCPro variants were agroinfiltrated together with a binary construct expressing GFP, as a reporter. In each leaf, we transiently expressed the reporter in a common leaf together with an empty vector, or together with the HCPro variants encoded by the Scottish and Spanish isolates as controls, as well as one Tunisian isolate, in order to eliminate any bias derived from use of different leaves. The six HCPros tested were able to suppress the partial silencing of the reporter in assays performed under either st or cc conditions (Figure 6). In assays performed under cc conditions, transient steady-state levels of the GFP reporter were lower than those under st conditions (Figure 6, lower leaf panels and associated Western blot to GFP panels vs. upper panels). None of the six HCPros assayed seemed to display silencing suppression strengths that were substantially different, relative to the others tested in the same leaf, under either growing condition (Figure 6).

## 4. Discussion

Plant viruses adapt to compatible hosts and vectors under specific climates [16,19,33]. On the one hand, climate change and global trade can facilitate the appearance of outbreaks of new viruses in crops and regions where they were not previously present. However, some plant virus species, on the other hand, do have natural global distributions, as is the case with some *Potyvirus* species, such as its type member PVY. Strains or isolates of cosmopolitan virus species would adapt to hosts/vectors in different geographies and climates [18,34]. In the case of *Potyviruses*, HCPro plays functions relevant to the systemic infection of compatible hosts, and to the horizontal dispersal of infections among plants [4,8]. HCPro could thus be an evolution target to achieve adaptation to local environments. In this regard, severe and mild natural strains in cucurbits of the potyvirus *Zucchini yellow mosaic virus* were found to be caused by a single variation in the FRNK motif of their HCPros (FRNK, severe→FINK, mild), associated with a dramatic drop in symptoms and titers [7]. On the other hand, there is evidence that sequences within other potyviral cistrons appear to be targets of positive evolution to facilitate adaptation, such as the *CP* of PVY, possibly through vectors [35,36]; or, very markedly, the *VPg* cistron of a turnip mosaic potyvirus that evolved through mechanical passages in different Arabidopsis accessions, and under different watering conditions. Preferential occurrence of mutations within a small 19 amino stretch of the *VPg* was observed in the evolved virus variants, although much less frequent mutations were also found in other cistrons, including *HCPro* [33].

To study whether HCPro could be an evolution target to achieve adaptation to local environments, we comparatively assessed the infection of an experimental compatible host by natural PVY isolates of different geographic and climatic origins (Scotland, Spain and Tunisia), under two controlled environments: one equivalent to standard laboratory room temperature (st condition), another one of elevated temperature and CO_2_ levels (cc condition). We simultaneously analyzed properties of their encoded HCPros, to assess if this protein could contribute to differences in virulence among the viral isolates in these experiments. Previous work with the Scottish isolate in this experimental host had shown that elevated temperatures induced significant drops in titers, and also lowered symptoms [26,28]. We did not know whether this outcome would be common to other PVY isolates, regardless of their origin, or whether the detrimental effect of cc conditions would be less marked or even absent in isolates originating from warmer climates (i.e., the Tunisian ones). 

The two European isolates, originating from Scotland and Southern Spain, had been sampled several years ago from potato and pepper plants, respectively. They both belong to the PVY^O^ strain, which was prevalent in Scotland at the time the Scottish isolate was sampled, but has been since gradually replaced in that land by PVY^N^-type strains, perhaps favored for their higher transmissibility [37]. The Scottish isolate remains aphid transmissible, but the Spanish isolate is non-aphid transmissible [21]. It is possible that the latter occurred because of ulterior mechanical passages through *Nicotiana* plants, as has been reported in another isolate [12]. All but one of the North African isolates belong to the PVY^NTN^-type. They were all sampled directly from potato crops except one, which was sampled from a pepper plant. We do not know about their transmissibility by aphids. However, Monastir crops originated from both certified and non-certified seed potatoes, while those from El Kef were all grown from certified seeds [38]. Therefore, at least infections in the latter site would have been aphid-transmitted to the plants from which these isolates were sampled. 

Sequencing of the *HCPro cistrons* of the isolates used in this study showed that they clustered phylogenetically in three branches, which correlated with the three countries of origin (Figure 1). Identities among the three branches hovered at around 90%. There was no further sub-grouping within the branch of the Tunisian isolates, despite them originating from two distinct sites in the country (Figure 1). The *HCPro* cistrons of the Tunisian isolates were remarkably similar, both at the nt and at the amino acid levels. Seven of the Tunisian isolates actually expressed identical HCPro proteins, even if they originated from either of the two sampling sites (Figure 2a). This suggests high homogeneities among viral populations of PVY-infected potato crops in the agricultural North of Tunisia. The one, or at most two, amino acid differences among the HCPros of the Tunisian isolates took place between amino acids 35 and 263 (of the 456 of the protein), which fall inside the N-terminal domain, involved in aphid transmission [5,12], and the Central domain involved in silencing suppression [6,28,29]; protein dimerization [39]; and the triggering of some HR responses in potato [32] (Figure 2b). Therefore, the HCPros encoded by the Tunisian isolates were either identical or had only one or two amino acid differences among each other. 

We determined the pathogenicity/virulence features of those isolates (virus titers and infection symptoms) under two experimental growing environments in *N. benthamiana* plants. Analysis of systemic viral titers was performed measuring both relative viral genomic RNA levels by RT-qPCR, and viral CP levels by Western blot. Since two sets of qPCR oligos were needed, one for the Scottish and the Spanish isolates, and another one for the Tunisian isolates, relative comparisons of genomic RNA levels could be established between the Scottish and Spanish isolates (Figure 3a) on the one hand, and among the Tunisian isolates on the other (Figure 3b), but not between the two groups. However, when comparing CP levels, all isolates could be directly compared among themselves (Figure 4). 

With regard to genomic viral RNA titers in systemic leaf tissues, we observed several-fold decreases in titers for all the isolates tested, in infections that took place under cc conditions relative to those under st conditions. Decreases ranged from ~three-fold (Tunisian isolate 59A) to more than ten-fold (i.e., Spanish isolate or Tunisian isolate MIE) (Figure 3). When we analyzed CP levels in the same infected tissues, we also found decreases, but less marked, ranging from 20% to around three-fold (Tunisian isolates 59A and MIE, respectively) (Figure 4). We do not know the cause of this difference in titer values when measuring genomic RNA levels or CP levels. RT-qPCR data reflect actual relative levels of viral RNA in the infected tissue. It could be that the Western blot system underestimates the amounts of CP because of saturation of the NBT/BCIP substrate on the protein band and its densitometry analysis. Alternatively, CP levels could actually remain relatively high in infected cells under cc conditions, despite much lower levels of genomic viral RNA, because of the slower turnover and higher stability of the viral protein. In any case, this difference in titers when measuring genomic vs. CP levels had been previously observed by del Toro et al. [26,28] for the Scottish isolate. It is interesting that all the isolates tested here displayed the same trend of decrease in titers under cc conditions, because it suggests that this is a feature common to the species, although differences with regard to the degrees of such decreases were observed among the Tunisian isolates. In interactions in other pathosystems and/or under different high temperature conditions, outcomes on viral accumulation can be different [40]. 

We also assessed the symptoms of infection induced in this experimental host by the isolates tested in plants kept under either controlled st or cc conditions. Symptoms at 10 dpi were classified into four categories according to their severity (Figure 5). Under st conditions, all the isolates tested induced either strong or very strong symptoms, with severe leaf curling and stunting, and in some cases, also the appearance of necrosis (Figure 5). However, we observed isolate-specific differences with regard to the symptoms when considering both st and cc conditions (Figure 5). The Scottish and Spanish isolates, as well as some of the Tunisian isolates, induced mild symptoms under cc conditions, while other Tunisian isolates induced strong or very strong symptoms under cc conditions. Interestingly, these differences occurred despite very high similarities among the HCPros expressed by the Tunisian isolates. In fact, four Tunisian isolates expressing identical HCPros displayed different symptom categories (Figure 5; isolates 41, 59A, Kef 7 and Kef 11, indicated by arrows). Therefore, even though some Tunisian isolates encoded identical HCPro proteins, the symptoms they induced under cc conditions were different. 

We finally tested the relative strengths of the HCPros expressed by six of the isolates (from the Scottish and Spanish ones, and from Tunisian isolates 41, 59A, MIE and Kef Pep2) to suppress the silencing of a reporter in agropatch assays, performed under either environment condition (Figure 6). We found that the increase in steady-state levels of the GFP reporter in the presence of the suppressors was similar in the six cases, suggesting that any amino acid differences in their sequences did not affect their comparative abilities to suppress silencing in this host, under the environment conditions assessed. 

In conclusion, our data show that in our experimental system, differences in virulence observed among the natural PVY isolates of different geographical origins when infecting this common host under two controlled growing environments cannot be determined solely by their HCPro factors, and therefore, suggest that other viral factors or combinations of viral factors may be responsible for the observed differences.

## Figures and Tables

**Table 1:** Oligonucleotides used for the RT-PCR amplification of the *HCPro* cistrons from the PVY isolates that were used in this work and for the relative quantitation of viral levels by RT-qPCR. Added *BamH*I, *Sac*I or *Kpn*I restriction sites are highlighted in red. Met-Ala-encoding triplets were added upstream the triplet encoding the starting N-terminal HCPro Ser, to enhance translatability and are highlighted in black. A stop codon (GAT) was also added downstream of the protein ORF, and is also highlighted in black (CTA, complementary sequence).

Oligo Purpose	Name	For PVY Isolates	Oligo Sequence
For RT (cDNA)	P3′ RT	All	TTCTTAACAACTTGCAACACCAT
For PCR (HCPro cloning, sequencing)	P5′ 580	Scottish	ATGGGATCCATGGCATCGAATGCTGATAATTTTTGGAAGGG
	P5′ 582	Spanish	ATGGGATCCATGGCATCAAATGCTGAGAATTTTTGGAAGGG
	P5′ 581	Tunisian	ATGGGATCCATGGCATCAAGCGCTGAAAGCTTTTGGAAGGG
	P3′ 392	Scottish, Spanish	CATCTGCAGGAGCTCCTAACCAACTCTATAATGTTTTATATC
	P3′ 591	Tunisian	CATGGTACCTAACCAACTCTATAGTGCTTAATGTCAG
For RT-qPCR (relative quantitation of viral levels)	qPCR-Scot-Spain Fw	Scottish, Spanish	CTGTGGGGACAAAGGGAGTA
	qPCR-Scot-Spain Rv	Scottish, Spanish	GGATGCTTGCGGATTTCATA
	qPCR-Tunis Fw	Tunisian	AGCTTGGAACCTGGCCAAC
	qPCR-Tunis Rv	Tunisian	TAGGCAGTTCTGCATCATG
	18SrRNA Fw		GCCCGTTGCTGCGATGATTC
	18SrRNA Rv		GCTGCCTTCCTTGGATGTGG

## Data Availability

Nucleotide and amino acid sequence data of the HCPro cistrons of the Tunisian isolates have been deposited at the GenBank repository (GenBank accession MW916783-MW916797). Accessions for each individual isolate are shown in Figure 1.

## Supplementary Materials

The following are available online at https://www.mdpi.com/article/10.3390/plants10061086/s1, Figure S1, climatic charts indicating average maximum and minimum temperatures throughout the year of the regions in the countries from which the potato virus Y (PVY) isolates used in this study originated.
