# Supplementary material for: Differences in Virulence among PVY Isolates of Different Geographical Origins When Infecting an Experimental Host under Two Growing Environments Are Not Determined by HCPro
Source: Plants (Basel). 2021 May 28;10(6):1086. doi: 10.3390/plants10061086 (PMC8228399; doi:10.3390/plants10061086)

Figure S1. Climatic charts indicating average maximum and minimum temperatures throughout the year of the regions in the countries from which the potato virus Y (PVY) isolates used in this study originated. Upper charts, Scotland (Dundee-Angus region) and Spain (Málaga region), respectively; lower charts, the Monastir and El Kef regions where the Tunisian isolates were sampled.

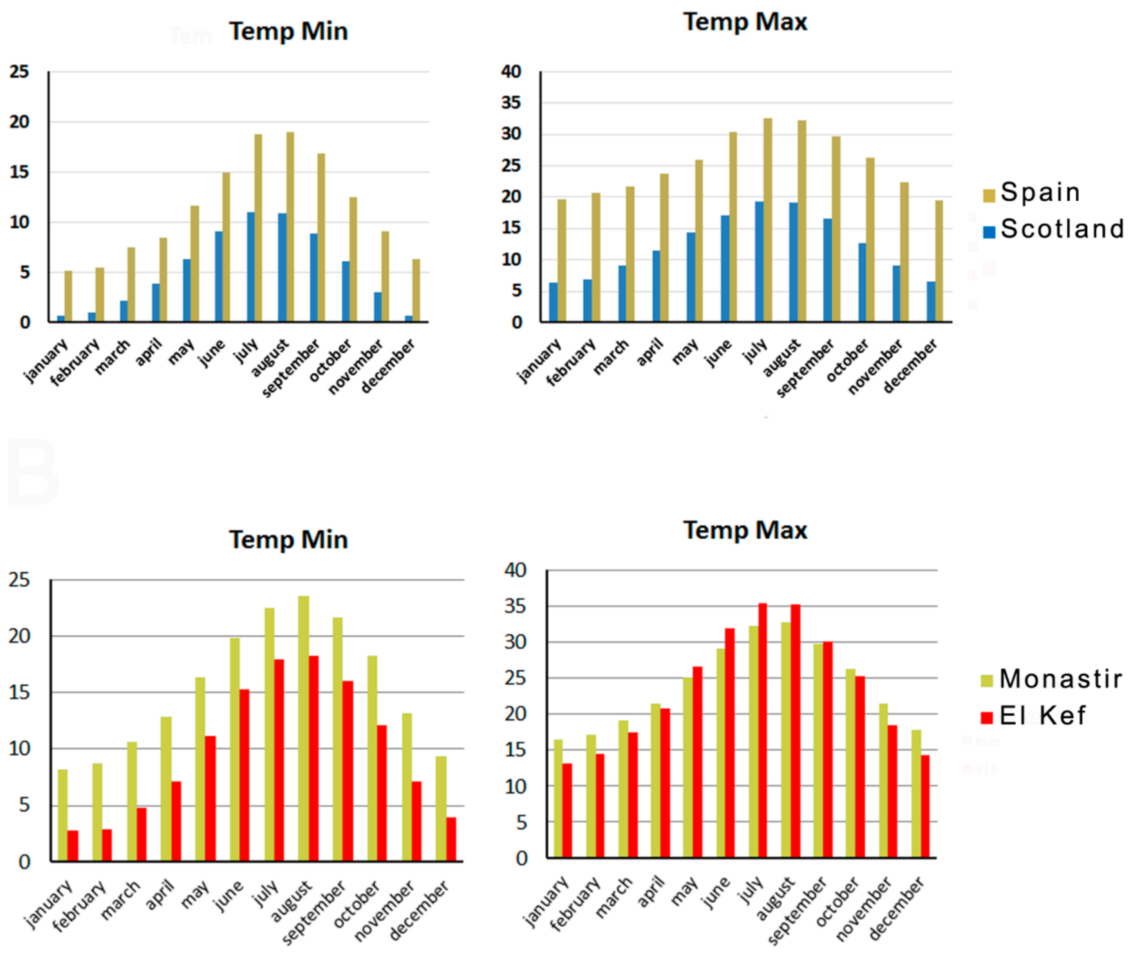

Supplement: Supplementary file 1 [file plants-10-01086-s001.zip › plants-1214385-supplementary.pdf]
